# Willpower as Cultural Construct: Do Chinese Students Believe Less in Its Depletion?

**DOI:** 10.3389/fpsyg.2019.00988

**Published:** 2019-05-03

**Authors:** Xin Sun, Kai S. Cortina, Kevin F. Miller, He Ning

**Affiliations:** ^1^Combined Program in Education and Psychology, University of Michigan, Ann Arbor, MI, United States; ^2^School of Psychology, Shaanxi Normal University, Xi’an, China

**Keywords:** belief about willpower, ego-depletion, cross-culture, East-West, China, United States

## Abstract

The idea of ego-depletion has been examined extensively in western cultures, but cultural background might substantially influence the understanding and effect of the concept. In the present study we used Job et al. (2010) Implicit Theories on Willpower Questionnaire to examine whether Chinese college students, compared to United States students, are less inclined to believe that willpower depletes. Applying two-group confirmatory structural equation modeling, the questionnaire with its two subscales – depletion of mental resources (DMR) and depletion of resistance to temptation – showed consistent psychometrical qualities across both samples. As predicted, Chinese student believed less in the concept of DMR than United States students. However, Chinese students showed a stronger belief in the depletion of resisting temptation (DRT) compared to their United States counterparts, suggesting different normative contexts for the response to the two subscales across cultures.

## Introduction

Concentrating on a cognitively challenging task for an extended time is a universal demand for college students. But after doing it for an hour, is it easier or harder for a student to concentrate on a similarly, demanding task? In another related scenario, if a person has been resisted eating a tasty-smelling cookie, would she be more likely to give up earlier on the subsequent problem-solving task than if she had not resisted a prior temptation? In their original studies, Baumeister et al. (1998) found that, after being asked to resist this kind of temptation, individuals persevered less on an immediately following problem-solving task. Generalizing from the initial experiments, they concluded that the demand for self-control impairs the cognitive performance of the subsequent task with similar cognitive loads. Baumeister proposed a *strength model of self-control*. He compared self-control with a muscle: it has limited energy to control one’s behavior that depletes over time (*ego-depletion* or *depletion of willpower*, Muraven and Baumeister, 2000; Baumeister et al., 2007). This state of depletion was demonstrated for a wide array of tasks, ranging from impression management (Vohs et al., 2005), decision making (Pocheptsova et al., 2009), to vigilance performance (Smit et al., 2004). The metaphor extended not only to the idea of recovery after a period of rest (Baumeister et al., 1994), but also to the idea that willpower can be strengthened through regular training (e.g., Gailliot et al., 2007). Lurquin and Miyake (2017) criticized the metaphorical conceptualization the willpower depletion phenomenon that was based on a nearly tautological definition. While those authors’ points are well-taken vis-à-vis the recent replication failure (Hagger et al., 2016), we follow the critical idea purported by Job et al. (2010, 2015). It re-conceptualizes the original idea of ego depletion as a belief system, i.e., as a more or less reflected belief that willpower actually depletes.

### Depletion of Willpower as a Belief Construct

Job et al. (2010) demonstrated that individuals’ theories about willpower, i.e., whether a person is convinced that willpower is limited, affect the degree to which ego depletion occurs. In its extreme, this hypothesis would imply that ego depletion only happens to those subjects who believe in the depletion and does not exist for people who do not buy into the idea. In this case, depletion of willpower would be nothing more than a self-fulfilling prophecy. In a sample of college students, Job et al. (2010) first measured beliefs about willpower (Example item: “Resisting temptations makes you feel more vulnerable to the next temptations that come along”). Next, participants were assigned to two conditions to do either a cognitively strenuous task (depletion condition) or a non-depleting task (non-depletion condition). Afterward, both groups worked on a different depletion task. Subjects who showed little belief in the depletion of willpower showed. In fact, no difference in the performance of both tasks, but the performance of subjects who believed that willpower depletes performed significantly lower on the second task. This moderation effect was replicated in studies on eating behavior and procrastination (Job et al., 2010). Besides, students who hold non-limitation beliefs about willpower exhibit better self-regulation in time management facing demanding workloads and have higher grades (Job et al., 2015).

One significant result of the Job et al. (2010) paper is that people differ substantially in their beliefs about whether willpower is limited. But if beliefs moderate the ego-depletion effect, the effect cannot be considered universal (Norenzayan and Heine, 2005). The size of the effect will depend on a large number of factors that directly or indirectly affect the belief systems of individuals in the process of socialization. The purpose of this study was to investigate whether culture is a contributing factor in the variation of the belief in depletion of willpower given that culture plays a significant role in shaping people’s beliefs (Hui and Triandis, 1986).

### Depletion of Willpower as a Cultural Construct

Comparison of beliefs across diverse cultural contexts is a topic of high interests among psychologists beyond the discourse on ego depletion. Conceptually speaking, it is reasonable to assume that ego depletion beliefs are associated with ideologies about learning, which usually involves cognitive exertion (Miller et al., 2012). Specifically, there is a particular interest in differences between collectivist cultures, especially Confucianism-based cultures like the Chinese, and individualism which is the dominant culture in the United States. This contrast is informative because the two cultures hold distinctive beliefs about learning (Li, 2003; Li et al., 2014). In the Chinese culture, perseverance is seen as a rather universal virtue. As Hong (2001) succinctly put it, “*exertion of effort is a cultural norm*” in China. Fan (2000) listed 71 core Chinese values, which included bearing hardship, persistence, and patience. Li et al. (2008) described the Chinese learning model as “*determination, diligence, endurance of hardship, perseverance, concentration, and humility*.” Empirical studies support the notion that depletion of willpower may be a foreign concept to the Chinese culture. A classroom observation study, for example, showed that Chinese students are able to engage in class work longer than their American counterparts (Lan et al., 2009). Chinese children outperformed American children on a series of executive function tasks, which measured attention control and inhibition (Lan et al., 2011). In a cart sorting study of learning-related terms, Li (2003) identified differences in the conceptual maps for “learning” of Chinese and American individuals. These maps showed that the Chinese attach importance to effort concerning learning, whereas westerners address ability. Specifically, the percentage of terms related to hard work and persistence was 30% in the Chinese versus 2% in the United States sample. Given the nuanced cultural differences in beliefs about learning-related cognitions, it is likely that beliefs about willpower as an important motivational factor of learning also differ systematically across cultures.

### On Identifying Cultural Differences: Methodological Issues

Cross-cultural psychology compares differences in psychological characteristics and constructs across cultures. Cultural psychology distinguishes “etic” and “emic” approaches. The “etic” approach investigates the universality of psychological constructs (Norenzayan and Heine, 2005), while the “emic” approach emphasizes cultural specificity (Triandis and Marin, 1983). An etic approach in quantitative cross-cultural research requires that measurement equivalence for a construct can be established across cultures, which includes – but is not limited to – issues of item translation. It also implies the equivalence of the construct validity, i.e., that in both cultures the measure correlates similarly, highly with conceptually related constructs and low with separate ones.

Comparative research on cultural differences in ego-depletion beliefs is therefore etic in nature. We want to investigate whether members of one culture on average endorse certain statements associated with the constructs more than members of another culture after having established measurement and construct validity equivalence.

### Perseverance, Grit as Related Constructs

Individuals holding a non-depleting theory of willpower believe that they can continue doing more work even after working for a long time (Job et al., 2010). There is conceptual overlap with two established psychological constructs: perseverance and grit. People with high perseverance, according to Whiteside and Lynam (2001), can maintain their attention on a particular task for a long time, even if the task is “*difficult or boring* (p. 685).” Similarly, grit is defined as “*perseverance and passion for long-term goals* (Duckworth et al., 2007)” which is conceptually related to willpower. These constructs differ from the belief in willpower mainly in their conceptualization as relatively stable traits while the beliefs are considered malleable. Beliefs may remain stable if unchallenged or not reflected, but could also be altered by interventions.

### Research Question

**FIGURE 1:**
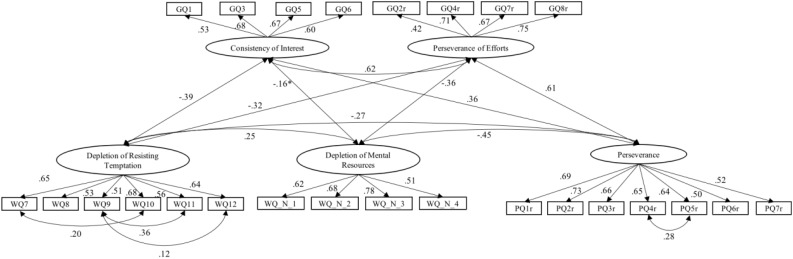
Structural model for the United States sample. All correlations are significant in a 0.0001 level except for the marked one; the marked one (with “^∗^”) indicates a significance at 0.05 level.

The current study aims to compare the belief in the depletion of willpower of Chinese and United States college students based on a psychometrically sound Chinese translation of Job et al. (2010) and Savani and Job (2017) questionnaire. To address the methodological issues on the cultural comparison, a structural equation model is tested to reflect the measurement of and the relationships among the three related constructs “belief in depletion of willpower,” “perseverance” and “grit” (Figure 1). If (and only if) measurement equivalence and construct validity across cultures can be established, we compare in a second step average beliefs in depletion of willpower of Chinese and United States college students as latent mean differences. We hypothesize that

(a) the questionnaire demonstrates qualified psychometric properties, which means it can be used to measure the same underlying construct in Chinese populations and
(b) Chinese, compared to their American counterparts, tend to embrace the idea of willpower depletion to a lesser degree; additionally, Chinese students are expected to show higher levels of perseverance and grit.

## Materials and Methods

### Participants

Participants were 803 college students, including 395 from a Midwestern university in the United States and 408 from two universities in northern and northwest China as part of a collaborative teaching project. All students were recruited from university psychology classes and were asked to complete an online Qualtrics questionnaire outside the classroom. Completion of the questionnaire was anonymous and voluntary; informed consent of the participants was implied through survey completion. Students were informed at the beginning that they could stop at any time without negative consequences. The study was granted exempt status by the Internal Review Board of the University of Michigan (HUM00098399, minimal risk). In the United States sample, 75.4% of the sample was female (with 0.3% indicating “other”), in the Chinese sample 74.3% (with 2% missing data). Around 98% of the students from the United States and 97% from China fell in the age range between 18 and 23.

### Measures

*Implicit theories on willpower (Job et al., 2010; Savani and Job, 2017):* In their research, Job et al. (2010, 2015) applied a 12-item questionnaire with two subscales measuring participants’ lay theory of depletion of willpower. It consists of two sub-scales, *depletion of resisting temptations* (DRT), and *depletion of mental resources* (DMR), each with six items answered on a 6-point Likert scale. A sample item of DRT is: “If you have just resisted a strong temptation, you feel strengthened and you can withstand any new temptations.” In their recent paper, Savani and Job (2017) revised the DMR sub-scale and developed a 4-item new version. In this version, each item consists of a scenario letting the participants imagine themselves concentrating on a cognitively demanding task. Then they are asked whether they think they could quickly restore their energy. For example, “Imagine you are working on very difficult math problems for 1 h. Do you believe that immediately after this, you would make more silly mistakes on another math test that also requires a lot of concentration, or would you make less silly mistakes on a difficult math test?” The new sub-scale is answered on a visually supported 20-point scale as developed in collaboration with the Indian research team to avoid the tendency to choose the middle category (Job, personal communication).

The current study adopted the DRT subscale from Job et al. (2010) and the DMR subscale from the refined version (Savani and Job, 2017) in order to maintain the option of a future comparison with that study. Of the 803 participants, all finished the DRT facet and 492 finished the DMR facet.

*The short grit scale (Grit-S, Duckworth and Quinn, 2009):* The construct of grit and its assessment was first discussed by Duckworth et al. (2007). The primary scale consisted of 12-items, 2-subscale construct, reduced by the original authors to an 8-item version which was used in this study. The two subscales are “Consistency of Interest” (GCI), e.g., “I often set a goal but later choose to pursue a different one,” and “Perseverance of Effort” (GPE), e.g., “I am a hard worker.” Items were responded on a 5-point scale from 1 (not like me at all) to 5 (very much like me).

*The perseverance scale (PER, Murray, 1938):* The perseverance scale was extracted from a set of personality measurements from Murray (1938). It has 7 items and items are rated on a 6-point scale ranging from 1 (strongly disagree) to 6 (strongly agree). E.g., “I can stand very long periods of exertion.”

### Procedure

The United States data were collected as an online survey at the beginning of the academic year in a junior level educational psychology lecture class in preparation of a lesson on international comparisons that utilized the data. The Chinese data were also collected online as part of an educational psychology class. Students who completed the questionnaire received 10 RMB (about 1.5 USD) as compensation. It took about 20–30 min to finish the questionnaire including demographics.

## Results

### Basic Psychometric Properties

See Table 1 for descriptive statistics as well as reliability estimates (Cronbach’s α) for each (sub-) scale. In general, all scales had a moderate to high reliability (ranging from 0.69 to 0.83). Table 2 shows the correlations among all (sub-) scales. As predicted, belief in depletion of willpower had significant negative correlations with perseverance and overall grit. Individuals who have lower levels of perseverance or grit are associated with holding a belief of limited willpower (DMR) sub-scale (*r* = −0.17 with grit; *r* = −0.34 with perseverance), and *r* = −0.33 and *r* = −0.20 with DRT sub-scale, all α < 0.01). One non-significant exception existed in the grit sub-scale of GCI, whose correlation with DMR was *r* = –0.06. Perseverance (PER) had a stronger association with the GPE subscale of grit (*r* = 0.44, *p* < 0.01) compared to the correlation with GCI (*r* = 0.21, *p* < 0.01).

**Table 1:** Descriptive Statistics.

	United States			China		
	Mean	SD	α	Mean	SD	α
DMR	12.23	4.08	0.76	10.36	4.70	0.77
DRT	3.13	0.77	0.78	3.34	0.77	0.70
GRIT	3.47	0.57	0.79	3.15	0.59	0.77
GPE	3.81	0.64	0.69	3.36	0.69	0.70
GCI	3.16	0.73	0.73	2.87	0.74	0.72
PER	3.49	0.85	0.79	3.62	0.85	0.83

*DMR, Depletion of Mental Resources; DRT, Depletion of Resisting Temptation; GRIT, Grit Scale, GCI, Consistency of Interests (subscale of Grit); GPE, Perseverance of Efforts (subscale of Grit); PER, Perseverance.*

**Table 2:** Correlation among Scales.

	DMR	DRT	GRIT	GPE	GCI	PER
DMR	1.00					
DRT	0.19^∗∗^	1.00				
GRIT	−0.17^∗∗^	−0.33^∗∗^	1.00			
GPE	−0.16^∗∗^	−0.28^∗∗^	0.89^∗∗^	1.00		
GCI	−0.06	−0.28^∗∗^	0.82^∗∗^	0.53^∗∗^	1.00	
PER	−0.34^∗∗^	−0.20^∗∗^	0.43^∗∗^	0.44^∗∗^	0.21^∗∗^	1.00

*^∗∗^indicates α < 0.01.*

### Measurement Invariance Test: Multi-Group Structural Model

To test more in depth whether the measures have the same meaning among Chinese and the United States students, a multiple-sample latent confirmatory analysis was conducted in order to establish measurement equivalence. Kline (2016) suggested four levels of measurement invariance: (a) configural invariance, (b) weak invariance, (c) strong invariance and (d) strict invariance. Configural invariance holds that the same confirmatory factor model (CFA) exists in both populations, allowing free estimation of all parameters across groups. Weak invariance adds an equality constraint over the unstandardized regression coefficients of the factor loadings of every parameter. Strong invariance, in addition, assumes equal unstandardized intercepts; strict invariance, in addition to all of the above, requires equal error variances and covariates across samples (Kline, 2016). We compared four models, beginning with the most parsimonious type, strict invariance, successively allowing for more parameters to vary between the two samples.

The goodness of fit indices (Table 3) rejected the model of strict measurement invariance (Model 1) with an RMSEA of 0.066, CFI 0.60, TLI 0.80, and SRMR 0.091. As the modification indices suggested, we allowed four within-construct error co-variances (Model 2), three within the belief in depletion of willpower and one for the perseverance scale. The model fit improved (RMSEA = 0.061, CFI = 0.83, TLI = 0.83, and SRMR = 0.088) but did not meet out target fit (TLI > 0.9). Model 3 removed some constraints of intercept equivalence (six from belief in willpower, one from grit and two from perseverance). The result showed a satisfactory overall fit (RMSEA = 0.057, CFI = 0.89, TLI = 0.89, and SRMR = 0.078). Model 4, intercepts of fourteen out of twenty-five items were unconstrained which improved the fit slightly (RMSEA = 0.048, CFI = 0.90 TLI = 0.89, and SRMR = 0.07). We adopted the more parsimonious Model 3 which demonstrated partially strong measurement invariance and fully met the criteria of weak invariance.

**Table 3:** Goodness of Fit Indices of the Measurement Invariance Models.

Model	Chi-square	*df*	Chi-square/*df*	RMSEA	CFI	TLI	SRMR
1	1644.47	605	2.72	0.066 (0.062,0.070)	0.80	0.80	0.091
2	1486.50	597	2.49	0.061 (0.057,0.065)	0.83	0.83	0.088
3	1359.53	586	2.32	0.057 (0.053,0.061)	0.85	0.85	0.078
4	1101.80	572	1.93	0.048 (0.044,0.052)	0.90	0.89	0.070

To test for equivalent construct validity regarding related construct (perseverance, grit), we tested the hypothesis of equality of the (latent) covariance matrix across groups. The χ*^2^* -difference test was not significant (χ*^2^* = 14.36, *df* = 30, *p* > 0.50) supporting the assumption of equivalence across groups.

Detailed standardized estimates are shown in Figure 1, 2. In general, items exhibited moderate to high factor loadings (from 0.41 to 0.78). Loadings were similar across countries. Nearly all correlations among latent traits were significant at *p* < 0.0001 level. Similar constructs demonstrated relatively high correlation, e.g., DRT is closely associated with GCI (*r* = −0.42 in the Chinese sample and *r* = −0.39 in the United States sample). The other three constructs, DMR, PER, and GPE were also highly inter-correlated (*r*s ranging from −0.31 to 0.67).

**FIGURE 2:**
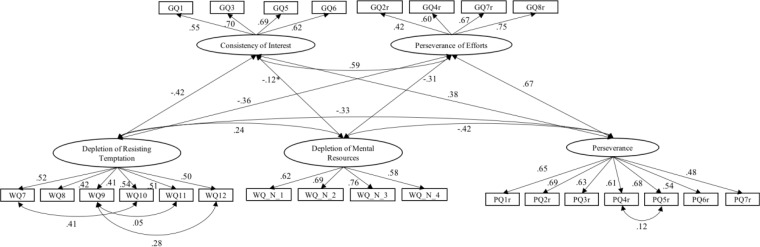
Structural model for the Chinese sample. All correlations are significant in a 0.0001 level except for the marked one; the marked one (with “^∗^”) indicates a significance at 0.05 level.

Latent factor mean differences (κ) of the four models are provided in Table 4. All models produce almost identical results, i.e., were robust with regard to the measurement invariance constraints. The two countries were significantly different on both subscales of belief in depletion of willpower. However, the two scale means differed in opposite directions.

**Table 4:** Latent Mean Differences (κ)of Two Countries for Each Scale for the Four Models.

		Model 1	Model 2	Model 3	Model 4
DMR	Mean Difference	−0.416	−0.419	−0.429	−0.351
	SE	0.096	0.097	0.098	0.099
	Significant	0.000	0.000	0.000	0.000
DRT	Mean Difference	0.305	0.314	0.341	0.331
	SE	0.083	0.093	0.099	0.116
	Significant	0.000	0.001	0.001	0.004
GCI	Mean Difference	−0.459	−0.459	−0.459	−0.426
	SE	0.083	0.083	0.084	0.102
	Significant	0.000	0.000	0.000	0.000
GPE	Mean Difference	−0.834	−0.835	−0.839	−0.809
	SE	0.088	0.088	0.089	0.093
	Significant	0.000	0.000	0.000	0.000
PER	Mean Difference	0.155	0.140	0.138	0.103
	SE	0.084	0.084	0.084	0.090
	Significant	0.065	0.098	0.099	0.252

For DMR, the United States students scored higher, which was predicted (κ = –0.351, *p* < 0.0001), meaning that the United States student, compared to their Chinese counterparts, tended to believe more strongly that one’s mental resources are limited and deplete. On the dimension DRT, however, the Chinese students were more prone to believe in the limited-resource model, contradicting our expectation (κ = 0.331, *p* < 0.0001). As for the grit scale, both dimensions demonstrated a higher level in the United States sample comparing to the Chinese with a κ = −0.426 (*p* < 0.0001) for GCI and κ = −0.809 (*p* < 0.0001) for GPE. The perseverance scale showed no significant difference between groups, with a κ = 0.103 (*p* = 0.252).

To address the potential effect from the differences in the gender ratio in the two samples, a multi-group comparison is conducted with gender as the grouping variable. Two models were compared: one with fixed loadings and covariances of all the latent construct and one where those were freely estimated. The χ*^2^*-difference is 46.30 with *df* = 30, *p* > 0.01. We retained the assumption of model equivalence across gender.

## Discussion

In the present study, we adopted the approach from Job et al. (2010) and Savani and Job (2017) to measure implicit theories about willpower to compare a United States and a Chinese student sample. Construct validity across cultures was confirmed by structural equation modeling including two other theoretically close latent variables, grit, and perseverance. We were able to document weak to strong measurement equivalence for the Chinese translations of the English scales and similar convergent validity in both cultures. The measurement invariance analysis confirmed overall generalizability of the beliefs in depletion of willpower as a construct across the boundaries of western individualistic cultures. As expected, Chinese college students tend to believe that their mental energy does not deplete, whereas the majority of the United States sample endorsed a view of limited resources. However, contrary to our prediction, the Chinese students showed an opposite pattern for the second component, belief in the DRT, which they endorsed on average more than the United States students.

The fact that the two samples show the expected differences in their beliefs in depletion of mental recourses but not resisting temptation leaves room for speculation regarding the cause for this interaction. East-Asian cultures particularly emphasize diligence and hardship in learning-related contexts (Li, 2003; Li et al., 2014). Endorsing an ego-depletion concept for cognitive demands may run counter to a strong normative belief that is part of a shared cultural identity while in the United States it is more widely accepted, even among college students, to have limited perseverance when it comes to applying cognitive resources. Resisting temptations on the other hand – according to Baumeister et al. (2007) equivalent to “*overcoming unwanted impulses*,” is not related to learning-relevant beliefs or practices and hence not a salient aspect of Asian students’ identity. Arguably more so than for the majority of young Chinese, growing up in the United States for many youths require navigating a demand-inducing market economy, which might lead to better developed coping strategies to resisting the everyday temptations of the consumer industry. It would be worthwhile for a further understanding of these results to explore in a more qualitative oriented study what kind of temptations the students in both cultures associate when they respond to the questions of the scale.

In general, our study corroborates the findings of the studies of Savani and Job (2017) who found that Indian individuals not only reject the idea of DMR, but also believe – in line with similar cultural stereotypes – that they would feel more energized after involving in a cognitively demanding task. These findings and the findings of our study suggest that substantial cultural differences exist in beliefs about ego-depletion but also in the way they are associated with actual behavior. At least for the example of the Chinese version of the belief in the depletion questionnaire, we are optimistic that comparative studies based on an emic research paradigm are possible with measurement instruments in translated versions.

Our study also underscores the domain-specificity of the ego-depletion effect and suggests restraint with generalizations beyond the specific task or belief under investigation. As Lurquin and Miyake (2017) argued, there are various tasks measuring self-control without clear validity. For example, resisting the temptation to eat mouth-watering cookies (DRT) and perseverance in working on challenging mathematics problems (DMR) may look at first glance as equivalent operationalization of the same construct, namely belief in ego-depletion but they may address qualitatively distinct kinds of “depletion” that can vary relatively independent of each other. Our study demonstrated only low correlations between the two scales (DRT and DMR) within each sample (United States: *r* = 0.25, China: *r* = 0.24) suggesting that, the concept of self-control or ego-depletion may not be as domain-general as it was initially proposed (Baumeister et al., 2007). The metaphor of energy depletion (or muscle that tires) would only work if resistance to temptation and cognitive task perseverance were jointly energized. As Lurquin and Miyake (2017) suggested, there could be well-designed correlational studies to show the underlying common component among different self-control tasks.

### Limitations

This study started off with the methodological challenge of translating psychological construct measures into Chinese meeting psychometrical standards and using them to directly compare mean scores for comparable populations in two different cultures. However, we measured beliefs and not actual behavior. Savani and Job (2017) measured beliefs and observed behavior, but did not translate the English scales and did not use the resisting of temptation subscale. We also need to acknowledge the fact that the student samples may not represent the general public of the two countries or cultural region. Additionally, although we did not find significant differences between sexes, this too may not well represent the general population. We do believe, however, that the college student populations at both sites are comparable concerning their positive selection regarding cognitive skills and academic success.

Depletion of willpower – or at least the belief in it – is a psychological construct that has the potential to become an essential piece in understanding cultural differences including learning behaviors and would provide a missing link between intercultural differences in more abstract qualities like field dependence (e.g., Kitayama et al., 2000) and differences in learning outcomes (Sellar and Lingard, 2013).

## Ethics Statement

This study was exempt from further review of the Institutional Review Board based on the fact that it is based on an voluntary and anonymous online questionnaire.

## Author Contributions

KC conceptualized the study, co-organized the data collection, and wrote the result section. XS implemented the questionnaire and wrote the first draft of the introduction. KM co-organized the data collection in the United States and in Beijing and reviewed the manuscript. HN organized data collection in Xi’an, China and reviewed to result and discussion section.

## Conflict of Interest Statement

The authors declare that the research was conducted in the absence of any commercial or financial relationships that could be construed as a potential conflict of interest.

## References

[B1] Baumeister R. F. Bratslavsky E. Muraven M. Tice D. M. (1998). Ego depletion: is the active self a limited resource? *J. Pers. Soc. Psychol.* 74 1252–1265. 9599441 10.1037//0022-3514.74.5.1252

[B2] Baumeister R F. Heatherton T. F. Tice D. M. (1994). *Losing Control: How and Why People Fail at Self-regulation.* San Diego, CA: Academic Press.

[B3] Baumeister R. F. Vohs K. D. Tice D. M. (2007). The strength model of self-control. *Curr. Dir. Psychol. Sci.* 16 351–355.

[B4] Duckworth A. L. Peterson C. Matthews M. D. Kelly D. R. (2007). Grit: perseverance and passion for long-term goals. *J. Pers. Soc. Psychol.* 92 1087–1101. 17547490 10.1037/0022-3514.92.6.1087

[B5] Duckworth A. L. Quinn P. D. (2009). Development and validation of the short grit scale (GRIT–S). *J. Pers. Assess.* 91 166–174. 19205937 10.1080/00223890802634290

[B6] Fan Y. (2000). A classification of Chinese culture. *Cross Cult. Manage.* 7 3–10.

[B7] Gailliot M. T. Plant E. A. Butz D. A. Baumeister R. F. (2007). Increasing self-regulatory strength can reduce the depleting effect of suppressing stereotypes. *Pers. Soc. Psychol. Bull.* 33 281–294. 17259587 10.1177/0146167206296101

[B8] Hagger M. S. Chatzisarantis N. L. Alberts H. Anggono C. O. Batailler C. Birt A. R. (2016). A multilab preregistered replication of the ego-depletion effect. *Perspect. Psychol. Sci.* 11 546–573. 27474142 10.1177/1745691616652873

[B9] Hong Y. (2001). “Chinese students’ and teachers’ inferences of effort and ability,” in *Student Motivation: The Culture and Context of Learning*, eds Salili F. Chiu C. Hong Y. (New York, NY: Kluwer/Plemum), 105–120.

[B10] Hui C. H. Triandis H. C. (1986). Individualism-collectivism: a study of cross-cultural researchers. *J. Cross Cult. Psychol.* 17 225–248.

[B11] Job V. Dweck C. S. Walton G. M. (2010). Ego depletion—Is it all in your head? implicit theories about willpower affect self-regulation. *Psychol. Sci.* 21 1686–1693. 10.1177/0956797610384745 20876879

[B12] Job V. Walton G. M. Bernecker K. Dweck C. S. (2015). Implicit theories about willpower predict self-regulation and grades in everyday life. *J. Pers. Soc. Psychol.* 108 637–647. 10.1037/pspp0000014 25844577

[B13] Kitayama S. Markus H. R. Kurokawa M. (2000). Culture, emotion, and well-being: good feelings in Japan and the United States. *Cogn. Emot.* 14 93–124. 10.1080/026999300379003

[B14] Kline R. B. (2016). *Practice of Principles of Structural Equation Modeling.* Thousand Oaks, CA: Sage.

[B15] Lan X. Legare C. H. Ponitz C. C. Li S. Morrison F. J. (2011). Investigating the links between the subcomponents of executive function and academic achievement: a cross-cultural analysis of Chinese and American preschoolers. *J. Exp. Child Psychol.* 108 677–692. 10.1016/j.jecp.2010.11.001 21238977

[B16] Lan X. Ponitz C. C. Miller K. F. Li S. Cortina K. Perry M. (2009). Keeping their attention: classroom practices associated with behavioral engagement in first grade mathematics classes in China and the United States. *Early Childh. Res. Quart.* 24 198–211.

[B17] Li J. (2003). US and Chinese cultural beliefs about learning. *J. Educ. Psychol.* 95 258–267.

[B18] Li J. Fung H. Bakeman R. Rae K. Wei W. (2014). How European American and Taiwanese mothers talk to their children about learning. *Child Dev.* 85 1206–1221. 10.1111/cdev.12172 24116837

[B19] Li J. Holloway S. D. Bempechat J. Loh E. (2008). Building and using a social network: nurture for low-income Chinese American adolescents’ learning. *New Dir. Child Adolesc. Dev.* 2008 9–25. 10.1002/cd.220 18792948

[B20] Lurquin J. H. Miyake A. (2017). Challenges to ego-depletion research go beyond the replication crisis: a need for tackling the conceptual crisis. *Front. Psychol.* 8:568. 10.3389/fpsyg.2017.00568 28458647 PMC5394171

[B21] Miller E. M. Walton G. M. Dweck C. S. Job V. Trzesniewski K. H. McClure S. M. (2012). Theories of willpower affect sustained learning. *PLoS One* 7:e38680. 10.1371/journal.pone.0038680 22745675 PMC3382137

[B22] Muraven M. Baumeister R. F. (2000). Self-regulation and depletion of limited resources: does self-control resemble a muscle? *Psychol. Bull.* 126 247–259. 10748642 10.1037/0033-2909.126.2.247

[B23] Murray H. A. (1938). *Explorations in Personality.* New York, NY: Oxford University Press.

[B24] Norenzayan A. Heine S. (2005). Psychological universals: what are they and how can we know? *Psychol. Bull.* 131 763–784. 16187859 10.1037/0033-2909.131.5.763

[B25] Pocheptsova A. Amir O. Dhar R. Baumeister R. F. (2009). Deciding without resources: resource depletion and choice in context. *J. Market. Res.* 46 344–355.

[B26] Savani K. Job V. (2017). Reverse ego-depletion: acts of self-control can improve subsequent performance in Indian cultural contexts. *J. Pers. Soc. Psychol.* 113 589–607. 10.1037/pspi0000099 28581300

[B27] Sellar S. Lingard B. (2013). Looking East: Shanghai, PISA 2009 and the reconstitution of reference societies in the global education policy field. *Comp. Educ.* 49 464–485.

[B28] Smit A. S. Eling P. A. Coenen A. M. (2004). Mental effort causes vigilance decrease due to resource depletion. *Acta Psychol.* 115 35–42. 14734240 10.1016/j.actpsy.2003.11.001

[B29] Triandis H. C. Marin G. (1983). Etic plus emic versus pseudoetic: a test of a basic assumption of contemporary cross-cultural psychology. *J. Cross Cult. Psychol.* 14 489–500.

[B30] Vohs K. D. Baumeister R. F. Ciarocco N. J. (2005). Self-regulation and self-presentation: regulatory resource depletion impairs impression management and effortful self-presentation depletes regulatory resources. *J. Pers. Soc. Psychol.* 88 632–657. 15796665 10.1037/0022-3514.88.4.632

[B31] Whiteside S. P. Lynam D. R. (2001). The five-factor model and impulsivity: using a structural model of personality to understand impulsivity. *Pers. Individ. Differ.* 30 669–689.

